# Multiplexed activity metabolomics for isolation of filipin macrolides from a hypogean actinomycete

**DOI:** 10.1038/s41429-024-00792-6

**Published:** 2024-12-06

**Authors:** Jordan T. Froese, Joseph A. Balsamo, Benjamin J. Reisman, Sierra M. Barone, Jonathan M. Irish, Brian O. Bachmann

**Affiliations:** 1 Department of Chemistry, Ball State University, Muncie, IN USA; 2 Department of Pharmacology, Vanderbilt University, Vanderbilt University School of Medicine, Nashville, TN USA; 3 Program in Chemical & Physical Biology, Vanderbilt University School of Medicine, Nashville, TN USA; 4 Vanderbilt-Ingram Cancer Center, Vanderbilt University Medical Center, Nashville, TN USA; 5 Department of Cell & Developmental Biology, Vanderbilt University, Nashville, TN USA; 6 Department of Pathology, Microbiology, & Immunology, Vanderbilt University Medical Center, Nashville, TN USA; 7 Department of Chemistry, Vanderbilt University, Nashville, TN USA

**Keywords:** Natural product synthesis, Screening

## Abstract

Chemical and biological stimulus screening in a hypogean actinomycete was used to elicit secondary metabolism. Optimal biosynthesis of bioactive natural products was identified using Multiplexed Activity Profiling for determining dose-dependent activity via six single-cell biological readouts. Bioactive extracts were fractioned to establish candidate compounds for isolation using Multiplexed Activity Metabolomics by correlating microtiter well-isolated phenotypes and extracted ion current peaks. This guided the isolation of four filipin polyene macrolides including a new metabolite filipin XV, an alkyl side-chain hydroxylated congener of the filipin chainin, with substantially attenuated cytotoxicity. Filipin-specific cytotoxicity was confirmed using flow cytometry and fluorescence microscopy.

## Introduction

Microorganisms across taxa are prolific for their potential to produce multiple unique secondary metabolites per organism, a potential that can now be quantified by automated analysis of microbial genomic and metagenomic sequences [1], and the majority of which remain to be isolated [2]. However, activating the production of predicted secondary metabolite gene clusters in microbes, isolating metabolites, and determining their structures and biological functions remain rate-limiting steps in fully capitalizing on the genome-evidenced potential of microbes. An array of techniques substantially increases cryptic and relatively silent secondary metabolite biosynthesis in microorganisms and aid in their identification. Genetic refactoring in native organisms [3, 4] and/or heterologous expression in chassis organisms [5] is increasingly attempted, though this approach is sometimes limited by the availability of practicable genetic techniques for genomic manipulation in producing strains, potential complications in regulatory element compatibility, precursor supply, and metabolite toxicity in a given heterologous expressing chassis organism. For many years, simply varying production media compositions has yielded improvements in production of microbial secondary metabolites [6]. More recently, introducing discrete chemical and biological stimuli into a single medium can be used to identify conditions to upregulate production of secondary metabolites within metabolomes and to facilitate their identification via comparative metabolomics. These stimuli include culturing actinomycetes with sub-inhibitory concentrations of antibiotics [7, 8], toxic rare earth elements, mixed culture with competing/interacting organisms, and de-repression via selection of antibiotic resistance associated with transcription and translation apparatus. Development of high throughput metabolomic methodologies enabled screening of hundreds of stimulus conditions for activation of secondary metabolism in a given producer and comparative metabolomics has facilitated the rapid assessment of differences among perturbed systems to aid in identification of novel secondary metabolites in an activity-independent fashion [4, 9–11]. Through these methods, and providing sufficient effort, the products of the majority of previously cryptic gene clusters identified by genomic inference can be isolated.

In these workflows, determination of activity or biological function often occurs after the isolation of upregulated, sufficiently abundant, and chromatographically tractable metabolites. However, it is not uncommon that hard-won new metabolites lack identifiable activity, representing an inherent inefficiency in such workflows. To address the gap between abundance-guided discovery of variously activated secondary metabolism and determination of potential biological relevance before isolation, this work provides a methodology for discovery of bioactive secondary metabolites by measuring biological responses in single cells within the presence of chemically complex microbial metabolomes. The quantitative single cell chemical biological activity metabolomics [12] tools described herein leverage fluorescent flow cytometry to efficiently identify bioactive microbial extracts, metabolomic subfractions, and compounds therein that impact cell injury and cell death in human-derived cell lines. These assays, termed Multiplexed Activity Profiling (MAP) [13] and Multiplexed Activity Metabolomics (MAM) [14], evaluate single cell responses to metabolite challenge using fluorescent cell barcoding [15, 16] and fluorescently labeled antibody functional readouts. The MAP assay is used to screen for dose-dependent bioactivity in crude extracts while the MAM assay is used to fractionate bioactive extracts into a comprehensive metabolomic array to reveal the bioactive component(s) within the complex chemical mixture. Herein, this MAM platform performs 228 individual assays per extract metabolome comprising 40 fractionated wells, eight control wells, five cell functional readouts, and overall cytotoxicity. Chemically dependent responses in single cells are acquired using flow cytometry and the biological data are deconvoluted with the DebarcodeR algorithm to enhance and automate computation of biological activity [17]. This workflow can be used for diverse applications in the discovery of bioeffectors from virtually any source ranging from compound libraries to the products of primary and secondary metabolism.

To demonstrate this workflow herein, an actinomycete sourced from Blue Spring cave (Sparta, TN) was isolated by culturing with selective media [18, 19] and cultivated in the presence of multiple chemical and biological stimulus to optimize conditions for secondary metabolite biosynthesis [7, 9, 20, 21]. The resulting metabolomes were extracted to determine the efficacy of stimulus specific bioactivity in an array of serially diluted extracts with MAP. Follow up analysis of growth conditions possessing dilutable activity in extracts was performed using MAM to identify bioactive components for isolation and identification. Through this workflow, a family of natural products was identified that did not exhibit significant diagnostic shift in most intracellular cell injury markers but did show general cytotoxicity. Isolation and structure elucidation identified three chemically active compounds belonging to the filipin family as well as a novel filipin family metabolite not previously reported. Flow cytometry confirmed filipin cytotoxicity occurs independently of the apoptotic maker cleaved caspase (cCAS3) and fluorescent imaging illuminated that the known cholesterol mediated membrane-disrupting mechanism of filipin macrolides can be attenuated by hydroxylation of the pendant alkyl chain. The relatively quiescent intracellular marker response profiles assist defining single cell phenotypes diagnostic of identification of membrane active metabolites prior to isolation in future campaigns.

## Materials and methods

### Isolation of *K. psammotica ssp. carrieae* from cave sediments

Soil samples were collected from ten locations throughout the Blue Spring cave system located in White County, Tennessee, using sterile Falcon tubes and a sterile spatula. A 100 mg portion of the sediment sample was vortexed with 1-mL of sterile water, and the supernatant was serially diluted and plated on agar selective for actinobacteria. Agar plates were incubated at 30 °C for 1–3 weeks and screened for the presence of single actinobacteria colonies. *Kitasatospora psammotica ssp. carrieae* was obtained from loose, oligotrophic, rocky soil taken far from the cave entrance as one of 41 actinomycete isolates derived from cave sediments.

### Taxonomy of producing organism

The 16S rRNA sequence was amplified with 27F (AGA GTT TGA TCC TGG CTC AG) and 1525R (AAG GAG GTG ATC CAG CCG CA) universal primers, the resulting amplicon isolated (Qiagen QIAquick® Gel Extraction kit) and sequenced using the described primers. The resulting sequence was analyzed using the EZBioCloud database and NCBI database.

### Scanning electron microscopy

*K. psammotica ssp. carrieae* was plated on ISP-2 agar, overlaid with nylon membrane, and incubated at 30 °C for 3 weeks. Samples were air dried, coated with gold palladium using a Cressington 108 sputter coater and imaged on a FEI Quanta 250 SEM at 5 kV.

### Fermentation, extraction, and isolation

*Kitasatospora psammotica ssp. carrieae* was plated onto ISP-2 agar (yeast extract (Sigma) 4 g L^−1^, malt extract (Gibco) 10 g L^−1^, glucose (RPI) 4 g L^−1^, and agar (RPI) 20 g L^−1^, pH 7.2) from glycerol stocks, and incubated at 30 °C for 5–7 days. Cells were then harvested from solid culture, homogenized, and used to inoculate seed cultures (25-mL ISP-2 liquid media (yeast extract 4 g L^−1^, malt extract 10 g L^−1^, glucose 4 g L^−1^, pH 7.2) in 250-mL Erlenmeyer flasks). Seed cultures were incubated at 30 °C for 48 h, and cells were then harvested from liquid cultures, homogenized, and used to inoculate 500-mL ISP-2 media, ISP-2 + 1.5 mM LaCl_3_ (Fisher), or ISP-2 + 120 nM rifampin (Sigma) in 2-L fermentation flasks. 500-mL cultures were incubated at 30 °C for 7 days. LaCl_3_ and rifampin were added to respective cultures after overnight incubation in 2-L fermentation flasks. After 7-day incubation, 100-mL of activated Diaion® HP-20 resin (Supelco) / H_2_O slurry was added to each 50-mL flask, and flasks were incubated with shaking for 3 h. Cells/resin were then centrifuged (3700 × *g*, 30 min), the supernatant discarded, and the resin/cellular mass extracted with 200-mL methanol/100-mL Diaion® HP-20 resin (Supelco) used (incubation with shaking for 1 h). Cell mass/resin was centrifuged (3700 × *g*, 30 min), methanol extract removed, concentrated and stored, and the resin/cellular mass extracted with 200-mL acetone/100-mL HP-20 resin used (incubation with shaking for 1 h). The cell mass/resin was again centrifuged (3700 × *g*, 30 min), and the acetone extract was removed, concentrated, and stored. Crude acetone extract was fractionated with Sephadex LH-20 resin (GE Healthcare Bio-Sciences) with methanol as the eluent. Fractions were analyzed by analytical HPLC/MS, and fractions containing the compound(s) of interest were pooled and further purified by preparative HPLC (Waters, XBridge C18 Prep, 5 µM) using a gradient of solution A (H_2_O - MeCN (95:5), 10 mM NH_4_OAc) and solution B (H_2_O:MeCN (5:95), 10 mM NH_4_OAc) with a flow rate of 10 mL min^−1^ (0–1 min: 100% solution A, 5 min: 85% solution A; 15% solution B, 65 min: 15% solution A; 85% solution B, and 70 min: 100% solution B). In order to obtain analytical purity, fractions containing the compounds of interest (15 min, 18 min, 21 min, 23 min, 25 min) were pooled and purified by flash column chromatography (CH_2_Cl_2_ – MeOH (95:5) to CH_2_Cl_2_ – MeOH (80:20)). The structure of the filipins was elucidated using a combination of mass spectrometry and nuclear magnetic resonance spectroscopy data. Mass spectrometry data produced with electrospray ionization and collected in both positive and negative modes provided the molecular weight of the compound **3**—Theoretical: 649.3564 (M + Na), Observed: 649.3582 (M + Na), and compound **4** - Theoretical: 647.3771 (M + Na), Observed: 647.3770 (M + Na)). Correlated nuclear magnetic spectroscopy (COSY) allowed for the assignment of the spin systems present, multiplicity-edited heteronuclear single-quantum coherence spectroscopy allowed for assigned ^1^H shifts to be correlated to their corresponding ^13^C shifts, as well as for the assignment of shifts as corresponding to methylenes or methines. Full structure elucidation was completed with heteronuclear multiple-bond correlation spectroscopy, which allowed for the assignment of remaining shifts based upon their proximity to assigned shifts.

### Metabolomic array generation

Metabolomic array of crude of *K. psammotica ssp. carrieae* extract was prepared as described previously. Briefly, arrays were generated using a split flow 3:1 ratio with 3 parts to photodiode array detector and fraction collector: 1 part to TSQ Triple Quantum mass spectrometer. Fraction plates were prepared by injecting 20 μL of crude *K. psammotica ssp. carrieae* extract (1:1 methanol: water) via Thermo PAL auto-injector onto a Phenomenex Luna 5 µm C18(2) reverse-phase C_18_ HPLC column. The sample was fractionated using a gradient of 100% Buffer A (H_2_O – MeCN (95:5)) to 100% Buffer B (MeCN–H_2_O (95:5)) over 30 min at a flow rate of 1 mL min^−1^ and split in a 3:1 ratio with three parts going to the photodiode array detector and fraction collector and one part going to the MS reverse-phase HPLC column at a flow rate of 1 mL min^−1^. Fractions were collected in 1-min intervals in a 96 deep well plate. A volume of 150 µL of eluent from each well was transferred to 96 well plates and dried in vacuo using a Genevac HT-6 system according to the factory standard HPLC method.

### Emission and excitation profiles

Fluorescence profiles of 100 µL of 10 µM filipins were evaluated in a black v-bottom plate and acquired with a Biotek Synergy H4 Hybrid plate reader.

### Cell seeding and chemical challenge

Metabolomic array was seeded with 200,000 MV-4-11 cells/well and incubated overnight. Control wells were treated with 0.5% DMSO (3 wells) or natural product control staurosporine, etoposide, aphidicolin, rapamycin, or nocodazole in 200 µL. Following treatment of cells with natural product controls, vehicle or metabolomic fractions, 200 µl of cells (1 million cells/mL), were stained with 20 µL of Ax700 (Thermo A20010, stock concentration 20 μg mL^−1^) to a final concentration of 0.04 μg mL^−1^ 37 °C for 20 min at and protected from light. Cells were then transferred 96 well v-bottom polypropylene plate with 20 µL of 20% PFA (Alfa Aesar 47340) for a final PFA concentration of 1.6% for 10 min. at room temperature, and protected from light. The plate was centrifuged at 800 × *g* for 5 min, supernatant decanted by plate inversion and dabbed dry on a Kimwipe (Kimtech 34155). The plate was then vortexed, and cells permeabilized with 200 µL ice-cold MeOH for at least 20 min at −20 °C. After permeation and prior to fluorescent cell barcoding, cells were centrifuged at 800 × *g* for 5 min, decanted by inversion, vortexed, and resuspended in 200 µL PBS (Gibco 10010-023).

### Fluorescent cell barcoding

Eight 1:1.7 serial dilution stock concentrations of Pacific Blue (Thermo P10163) were prepared in DMSO at concentrations ranging from 10 µg mL^−1^ to 0.24 µg mL^−1^. Six 1:1.7 serial dilution stock concentrations of Pacific Orange (Thermo P30253) were prepared in DMSO at concentrations ranging from 40 µg mL^−1^ to 2.82 µg mL^−1^. Alexa750 (Thermo A20011) 500 µg mL^−1^ stock solution in DMSO was prepared at 5 µg mL^−1^. All three dyes were combined on a single V-bottom polypropylene master plate as follows: 50 µL of Pacific Blue were added to plate rows with the highest concentration in row A and the lowest concentration in row H; 50 µL of Pacific Orange was added to columns with the highest concentration in column 1 and lowest concentration in column 6; 50 µL of Alexa 750 were added to all wells. Primary plates were then aliquoted in 15 µL volumes to 96 well V-bottom propylene plates and stored at –20 °C. An aliquot of 185 µL of fix/permed cells in PBS was added to 15 µL of barcode dyes and stained for 15 min at room temperature, protected from light. Barcoding reactions were quenched with 70 µL 2% BSA in PBS, centrifuged at 800 × *g* for 5 min, decanted by inversion, vortexed, washed with 200 µL 2% BSA in PBS, centrifuged at 800 × *g* for 5 min, decanted by inversion, and vortexed. Wells in column 1 were then resuspended in 200 µL of 2% BSA in PBS then transferred to column 2 and repeated to column 6. All rows in column 6 were then pooled into 1 flow cytometry tube.

### Fluorescent cell staining

Cells were stained with antibodies γH2AX:Percp-Cy5.5 (BD 564718, 2.5:100), cCAS3:PE (10:100), p-HH3:Pe-Cy7 (Biolegend 6410, 1:1000), p-S6:Ax647 (CST. 4:100) for 30 min at 4 °C then washed with 2% BSA/PBS and resuspended in 475 μL of 2% BSA/PBS. The DNA intercalator YoPro (Invitrogen) was diluted 1:1000 in 960 μL of 2% BSA/PBS plus 40 μL of RNAse for addition of 25 μL to each stained experiment for a total volume of 500 μL.

### Flow cytometry

Flow cytometry data were acquired using a four laser BD fortessa. Acquired data were uploaded to Cytobank for compensation and gating. Samples were debarcoded with DebarcodeR to produce one .fcs file per well and uploaded to Cytobank for storage and further analysis.

### Fluorescent microscopy

MV411 cells were seeded on Greiner Bio-One black-glass bottom 96 well plates and challenged with each compound overnight. Cells were stained with 10 μM quinacrine for 30 min at room temperature in the dark and washed with 2% BSA/PBS. Cells were stained with 300 nM propidium iodide. Images were captured with ImageXpress Micro XL fluorescent microscope.

### Statistical analysis and figure generation

Data was input to GraphPad Prism (9.2.0) and analyzed with one-way ANOVA. All data is reported as mean ± standard deviation (S.D.) of at least 3 biological replicates. GraphPad Prism, Adobe Illustrator 2021, Microsoft Excel 2016 were used for figure generation.

### General experimental procedures

Mass spectrometry was performed by using a TSQ Triple Quantum mass spectrometer equipped with an electrospray ionization source and Surveyor PDA Plus detector (Thermo Fisher Scientific, Waltham, MA, USA) (ESI, positive and negative mode). High-Resolution Electrospray Ionization-Mass Spectrometry (HRESI-MS) was performed using a Waters Synapt G2S HDMS (Milford, MA, USA). NMR spectra were recorded on a 600 MHz Bruker AV-II FT NMR spectrometer with cryoprobe & LC-SPE (Bruker BioSpin Corp., Billerica, MA, USA). Methanol-*d*_4_ (Sigma-Aldrich) and dimethyl sulfoxide-*d*_6_ (Sigma-Aldrich) were used as solvents, and the chemical shifts were referenced to the solvent signal of methanol-*d*_4_ (3.30 ppm).

## Results

### Isolation of *Kitasatospora* from Blue Spring Cave

**Fig. 1:**
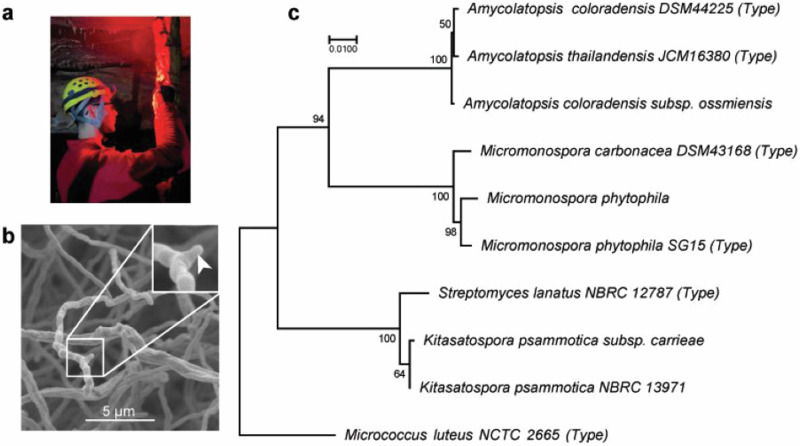
Caves are an ecological niche for new actinomycetes. (**a)** Passage within Blue Spring Cave. **(b)** Scanning electron micrograph of aerial hyphae and smooth tendrils with dull protrusions that lack clearly defined sporangia. White arrowhead in inset B indicates dull protrusion. **(c)** The evolutionary history was inferred by using the Maximum Likelihood method and Tamura-Nei model. The tree with the highest log likelihood (-3932.17) is shown. Initial tree(s) for the heuristic search were obtained automatically by applying Neighbor-Join and BioNJ algorithms to a matrix of pairwise distances estimated using the Maximum Composite Likelihood (MCL) approach, and then selecting the topology with superior log likelihood value. The tree is drawn to scale, with branch lengths measured in the number of substitutions per site. This analysis involved ten nucleotide sequences. There were a total of 1537 positions in the final dataset. Evolutionary analyses were conducted in MEGA 7

Actinobacteria were isolated from cave sediments obtained from Blue Spring Cave in White County, Tennessee (Fig. 1a) via dilution plating on agar media (See Methods). One putative actinobacterial strain developed into an orange lawn with white mycelia on ISP-2 agar and formed cylindrical aerial hyphae with smooth spore chains dotted with protruding nodules (Fig. 1b and Supplemenatary Fig. S1). The 16S ribosomal gene sequence was used to infer the likely evolutionary history of this species by bootstrapping 1000 maximum likelihood projections in MEGA 7 [22] of 16S ribosomal DNA sequence alignment with closest relatives predicted by EZBiocloud’s 16S-based [23] ID search algorithm and 16S sequences of closest type strains from *Streptomyces, Micromonospora*, and *Amycolatopsis* (Fig. 1c). EZBioCloud identified *Kitasatospora psammotica* (99.93% similarity, basonym *Streptomyces psammotica*) [24, 25] as the closest evolutionary relative consistent with the phylogenetic tree (Fig. 1c). The percent similarity of 16S DNA was given the subspecies (*ssp*.) designation *carrieae* (Fig. 1c) in recognition of Lonnie Carr, a long-standing cave conservationist and owner of Blue Spring Cave, generously providing access to this gated 30+ mile system to researchers and explorers for decades.

### Multiplexed Activity Profiling (MAP) affords high-throughput, dose-dependent interrogation of bioactive metabolites

**Fig. 2:**
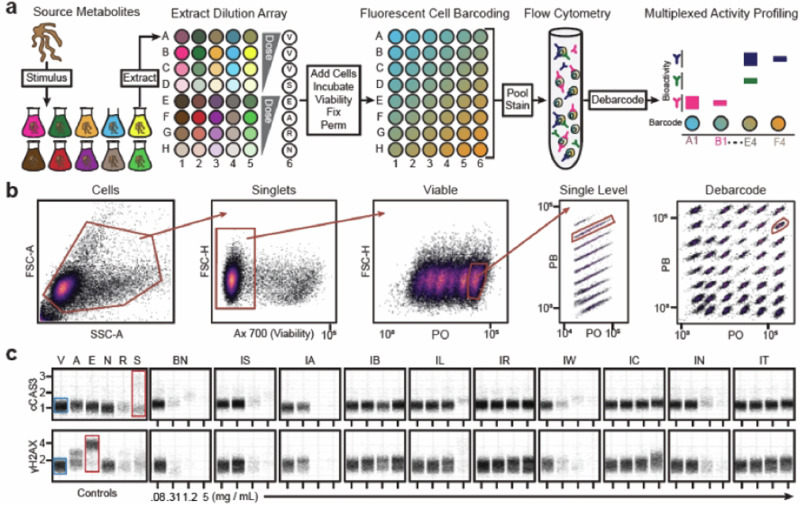
Multiplexed activity profile of single cells challenged with metabolomes from actinomycetes cultured in diverse stimuli. (**a**) Harvested actinomycetes were incubated in the presence of diverse stimuli and then secondary metabolites extracted for bioactivity dose response in MV-4-11 cell line. Following incubation, cells were viability stained with Ax700, fixed with PFA, permeabilized with methanol, and fluorescently barcoded. Fluorescent cell barcoding is used to conserve well position through processing and analysis with NHS-functionalized, primary amine reactive Pacific Blue and Pacific Orange fluorescent dyes. Eight concentrations of PB are arrayed with six concentrations of PO to create 48 unique fluorescent identities that encode each well position. Barcoded cells are pooled and stained with a fluorescent antibody cocktail to measure molecular readouts with flow cytometry. Cells are digitally reassigned to well position via fluorescent barcode with DebacodeR algorithm. Extracts, well position, fluorescent barcode, and molecular readouts are combined to produce a multiplexed activity profile to assess bioactivity. (**b**) Quality control gating scheme of MAP assay. Collected events were gated by forward and side scatter to identify cells, forward scatter height for single cells, and Ax700 for viability. Six concentrations of pacific orange are encoded in the viable, singlets cells and each pacific orange concentration encodes eight pacific blue concentrations to identify cell populations by fluorescent barcode. A single cell population is tracked by the red gates and is identified in the debarcoded cell populations. (**c**) Multiplexed activity profile of *S. psammotiucs ssp carrie* cultured in diverse stimuli. Each black dot represents a single cell response at the observed dose and are compared to specific positive control compounds (red box) to infer single cell marker shifts versus vehicle control (blue box). This organism produced a cytotoxic compound that did not increase the number of apoptotic cells measured as the arcsinh ratio of median fluorescent intensity versus vehicle control but exhibited cytotoxic effects visualized by decreased viable cell populations. V = Vehicle, A = Aphidicolin, E = Etoposide, N = Nocodazole, R = Rapamycin, S = Staurosporine, PB = Pacific Blue, PO = Pacific Orange, BN = Bennet’s media, IS = ISP2, IA = ISP2-agar, IB = ISP2-B. subtilus, IL = ISP2-LaCl3, IR = ISP2-Rifampin, IW = ISP2-*Rhodococcus wratislaviensis*, IC = ISP2-*Escherchia coli*, IN = ISP2-Streptomycin, IT = ISP2-*Tsukamorella pneumonia*

A MAP protocol was used to test a dilution series of crude metabolomic extracts from *K. psammotica* ssp*. carrieae* cultured with chemical and biological stimuli to identify dose-dependent responses of diverse mammalian cell status markers (Fig. 2a). *K. psammotica* ssp*. carrieae* was cultivated under ten distinct stimulus conditions, including solid and liquid media (ISP-2 and Bennett’s media), in the presence of competitive microorganisms (*B. subtills*, *R. wratislavensis*, *T. pneumonia* and *E. coli*), in the presence of sub-inhibitory concentrations of antibiotics (rifampin and streptomycin) and in the presence of lanthanide salts (LaCl_3_) [7, 9, 20, 21, 26]. The resultant metabolomes were extracted by adding hydrophobic resin HP-20 to cultures and extracting cells and resin with methanol, diluted 64-fold across four wells in microtiter plates, and evaporated to yield an extract dilution array (Fig. 2a). Metabolites were resuspended in cell culture media containing MV-4-11 AML cells at a density of 1 million mL^−1^ and incubated overnight for 16 h.

After incubation cells were stained with a fluorescent Alexa 700 (Ax700) dye to label non-viable cells and measure overt cytotoxicity, then fixed in paraformaldehyde to memorialize cell status, and finally permeabilized in methanol to enable fluorescent antibody and fluorescent dye infiltration for detection of intracellular targets including cCAS3 (apoptosis), γH2AX (DNA damage), DNA (cell cycle), p-S6 (translation) and p-Hh3 (proliferation). Cells were fluorescently barcoded with an array of *N*-hydroxysuccinimide ester-functionalized amine (NHS) reactive fluorescent dyes composed of eight concentrations of Pacific Blue (one per row, A-H) and six concentrations of Pacific Orange (one per column, 1-6) to yield 48 well-specific fluorescently labeled cell populations (Fig. 2a). The barcoded cells were pooled and stained with a fluorescent module composed of antibodies and a DNA intercalator to detect bioactivity. Barcode and marker signal intensity were then acquired simultaneously with flow cytometry (Fig. 2a). Following signal acquisition, intact, singlet cell populations were gated by forward and side scatter parameters then gated for viable, Ax700 negative cells (Fig. 2b). Cells were debarcoded with the DebarcodeR algorithm to separate cell populations by fluorescent intensity of each barcode dye for digital reassignment to the original plate well (Fig. 2b). Conventionally, non-viable cells are excluded from analysis due to inconsistent reactions with fluorescent NHS-dyes and fluorescent antibodies that confound debarcoding and overall data interpretation.

Cells were phenotypically evaluated by inspecting bioactivity trends to determine bioeffector potential of each extract. *K. psammotica* ssp*. carrieae* extracts markedly reduced viable cell populations with relatively low functional marker shifts cross-referenced to bioactivity observed in positive control conditions: Staurosporine – cCAS3, Etoposide – γH2AX, Aphidicolin – DNA (G1), Rapamycin – p-S6, Nocodazole – p-HH3, DNA (G2) (Fig. 2c). For example, metabolites harvested from ISP-2 liquid culture exhibited viable cell loss at 1.2 mg mL^−1^ and 5 mg mL^−1^ extract doses as a consequence non-viable cell removal (Fig. 2c, IS). An increase in cytotoxicity was observed in viable cells remaining in effected wells (e.g. Figure 2c, BN, IW) and demonstrated less fluorescent signal of cell status markers compared to staurosporine or etoposide controls (Fig. 2c). Moreover, extracts taken from various stimulus conditions did not invoke marker changes or overt cytotoxicity including co-culture with *B. subtilis* and exposure to sub-lethal doses of rifampin antibiotic (Fig. 2c, IB and IR). Taken together, selective stimulus can stimulate or decrease biosynthesis of cytotoxic agents by *K. psammotica ssp. carrieae* that permeabilized cell membranes within 16 h of challenge (Fig. 2c). Moreover, this effect was observed at extract doses as minimal as 0.31 mg mL^−1^ (Fig. 2c), which motivated the decision to isolate and elucidate the bioactive agent(s) from *K. psammotica* ssp*. carrieae*. It is worth noting that the control compound staurosporine elicits an apoptotic cCAS3 response that can exhibit diminished cell populations relative to other compound controls as apoptotic cells with advanced membrane degradation are labeled non-viable by Ax700 and excluded from analysis (Fig. 2c). Conversely, etoposide challenged cells exhibited robust γH2AX DNA damage response without concomitant cell loss due to membrane disruption (Fig. 2c).

### Multiplexed Activity Metabolomics (MAM) detects bioactivity in discrete metabolomic fractions

**Fig. 3:**
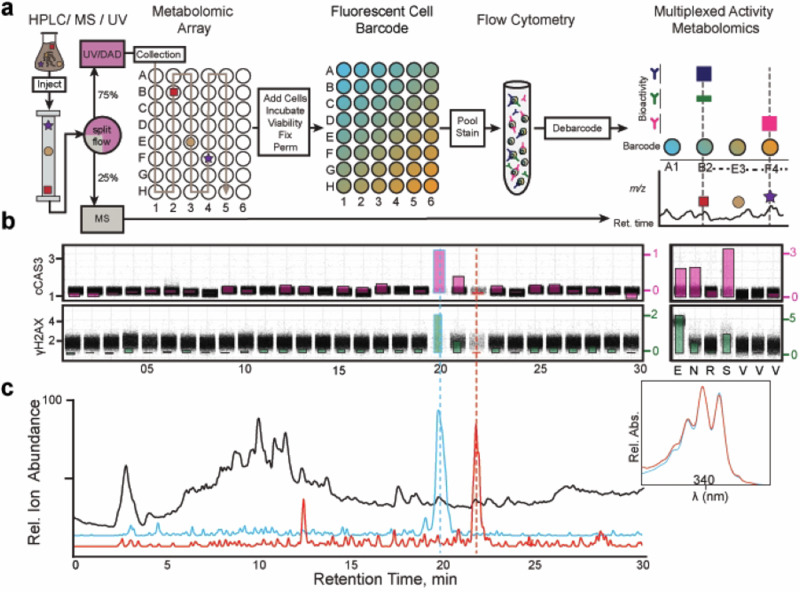
Multiplexed activity metabolomics identifies candidate bioactive compounds from single-cell data. (**a)** Whole metabolomes from active MAPs are fractionated into a metabolomic array with split flow (3:1) HPLC/UV/MS system. One-minute metabolomic fractions are collected in 48 wells, dried in vacuo, and resuspended in media-containing cells. Cells are incubated, fixed in PFA, permeabilized in methanol, fluorescently barcoded, pooled, stained, flowed, and debarcoded and compared to marker shifts and spectral data to determine well fractions with potential for isolating a bioactive secondary metabolite. **(b)** Each metabolomic fraction is evaluated independently for phenotypically disturbed cell populations. The single-cell response profile of *S. carrieae* metabolomic fractions reveals cell loss in fractions 20 and 22. (**c)** Active metabolomic fractions are cross-referenced against complementary mass spectral data (total ion current – orange trace) to identify candidate natural products for isolation. Candidate ion currents are extracted to observe temporal relationships to observed bioactivity. Extracted ion currents of 625.39 (blue trace) and 661.39 (red trace) highlight candidate bioactive compounds that elute at minutes 20 (blue trace) and 22, respectively

To identify the cytotoxic secondary metabolites, the *K. psammotica* ssp*. carrieae* extracted metabolome was fractioned by reverse phase chromatography connected to a split-flow polarity-switching electrospray mass spectrometric analyzer (ESI-MS) and a UV/VIS photodiode array detector (Fig. 3a). The ESI-MS portion is sacrificed to acquire mass spectra while the eluate from the UV/Vis is collected at a rate of 1 fraction min^−1^ of effluent per well to compare bioactive fractions with complementary spectral data and inform isolation of bioactive secondary metabolites. The *K. psammotica* ssp*. carrieae* metabolomic array was evaporated in microtiter plates *in vacuo* and resuspended in cell-containing media for incubation and subsequent processing with fluorescent cell barcoding, fluorescent staining, and debarcoding to identify well-specific bioactivity aligned with coordinated, discrete regions of the mass spectra (Fig. 3a and S2).

MV-4-11 cells incubated for 16 h with control compounds staurosporine and etoposide exhibited robust shifts for apoptotic and DNA damage markers respectively. Fraction-dependent responses at 16-h incubation were evaluated by comparison to positive and vehicle controls then quantified as the arcsinh ratio of percent cells positive for each marker verse vehicle control (Fig. 3b). While the fluorescent signal of cCAS3 and γH2AX were elevated in well 20 they were decreased in well 22 despite abundant cell death in each well (Fig. 3b). The lack of marker response for DNA, p-HH3, and p-S6 (Fig. S2) suggested signaling-independent cytotoxicity for these cellular processes. Overall cytotoxicity was determined by the percent viable cells in each well compared to vehicle control. Condition 20 and 22 demonstrated reductions of viable cells to 4.4% and 17.2% respectively compared to vehicle control (Fig. S3).

The bioactivity profiles were compared to liquid chromatography/mass spectrometry (LC/MS) data to identify and extract well-localized ion currents to observe relative abundance of metabolites for isolation. This identified two putative secondary metabolites with an *m/z* 625.4 in fraction 20 and *m/z* 639.4 in fraction 22 (Fig. 3c). The UV/Vis spectral data of these secondary metabolites demonstrated local UV maxima from 320–360 nm (Fig. 3c), consistent with the family of polyene antibiotics. Inspection for compounds with similar UV spectra identified two additional but less active polyenes in fractions 14 and 19 with positive extracted ion currents of *m/z* 627.4 and *m/z* 611.4 respectively (Fig. S3).

### Isolation and structural elucidation of filipins

**Fig. 4:**
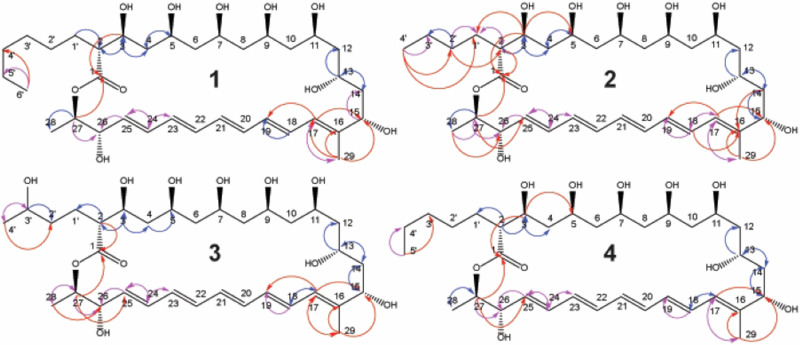
Structural assignment, exact mass, and spectral profiles of isolated filipin metabolites. (**1**) Filipin II; 639.41082 (**2**) Chainin; 611.37952 (**3**) Filipin XV; 627.37444 (**4**) Filipin IX; 625.39517. Blue = COSY correlation, Red = HMBC correlation, Purple = COSY and HMBC correlation

*K. psammotica* ssp*. carrieae* culture was scaled to afford isolation of the four polyenes. Cultures were extracted using HP-20 resin and metabolites purified via LH-20 chromatography and preparative HPLC. Established Fieser-Kuhn rules [27, 28] indicated that candidate *m/z* 639.4 likely contained five conjugated olefins. Further 1D NMR studies were also consistent with this metabolite containing multiple C-O methine functionality (Fig. S4 and Table S1). Based upon these parameters, literature searches identified the candidate compound as the known polyene macrolide metabolite filipin II(1), which was confirmed by subsequent NMR studies including correlated spectroscopy (COSY), heteronuclear multiple-bond correlated spectroscopy (HMBC), and heteronuclear single quantum coherence spectroscopy (HSQC) (Fig. 4, S4 and Table S1). The decreased mass of candidate *m/z* 611.4 indicated this metabolite lacked two methylene functional groups in comparison to the assigned structure of compound **1** (Fig. 4). Subsequent NMR analysis of this metabolite demonstrated the core filipin structure was intact, and that the pendant alkyl chain lacked two methylenes (Fig. 4, S5, and Table S2). These assignments confirmed the second polyene macrolide metabolite isolated as a the previously described member of the filipin family, chainin(2) (Fig. 4)[29, 30].

Following the identification of these previously reported members of the filipin family, two lower-abundance metabolites were isolated with *m/z* of 627.4 and 625.4 respectively. The *m/z* of the more polar of these metabolites (627.4) indicated the addition of a hydroxyl functional group relative to compound **2** (Fig. 4 and S6). In addition to sharing local UV maxima of 320–360 nM of the previously isolated filipin members, further analysis by 2D NMR revealed an intact filipin core and indicated an unusual hydroxylation pattern at carbon 3’ (**3** Fig. 4 and Table S3). This assignment, combined with high-resolution mass spectrometry analysis validated the molecular formula of this metabolite (theoretical: 649.3564 (M + Na), observed: 649.3582 (M + Na), and confirmed its identity as a previously unreported member of the filipin family herein referred to as filipin XV (**3**, Fig. 4) which was also light yellow in color. We were unable to selectively derivatize the C3’ for the purpose of resolving the stereochemistry. The final metabolite isolated possessed one additional methylene group relative to compound **2**, based upon its m/z of 625.4. 2D NMR analysis (COSY, HMBC, HSQC) of this metabolite demonstrated that the core filipin structure was intact, and that the additional methylene functionality was located on the pendant alkyl chain (Fig. 4, S7, and Table S4). The validation of the molecular formula of this metabolite (theoretical: 647.3771 (M + Na), observed: 647.3770 (M + Na)) confirmed the identity of this metabolite as filipin IX (**4**, Fig. 4).

### Filipin-induced cell permeation is caspase-3 independent

**Fig. 5:**
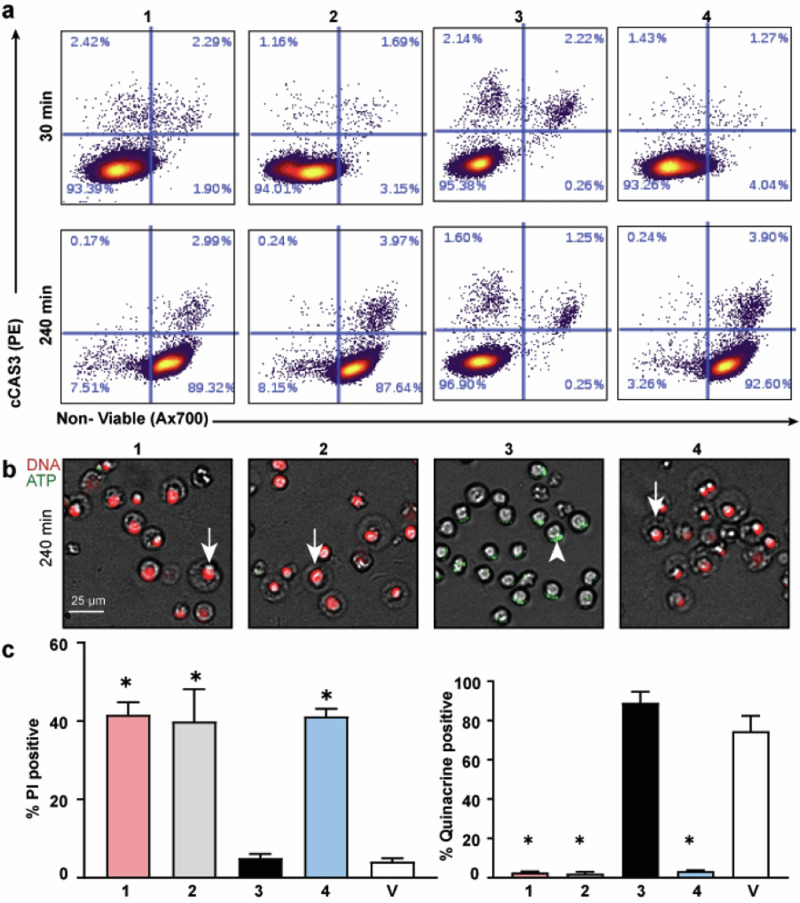
Filipin-induced death is cCAS3 independent and pendant alkyl chain dependent. **(a)** Single cell contour plots of apoptotic marker cCAS3 and Ax700 viability stain. Cells were treated for indicated time then stained with Ax700, fixed, permeabilized, and stained for cCAS3. Compounds 1, 2, and 4 disrupted cell membranes without activating cCAS3. **(b)** Cells were challenged with each filipin and stained with propidium iodide (cell viability, DNA) and quinacrine (intracellular ATP) cocktail. Compounds 1, 2, and 4 exhibit robust PI labeling (white arrows) and concomitant loss of ATP. Compound 3 does not enhance PI infiltration of loss of ATP (white arrowhead). (**c)** Quantification of percent positive PI or quinacrine positive cells versus vehicle control. Cells were challenged with 10 μM of each compound for the indicated time. *p < 0.05 vs vehicle control. Scale bar = 25 µm; Red, PI = DNA; Green, quinacrine = ATP

The staurosporine and filipin injury phenotypes shared similar losses of viable cells (Fig. S3). Since staurosporine-induced apoptosis also yields a degree of non-viable cell exclusion in this system (Fig. 3 and S3), it was possible that filipins engaged apoptosis to account for overall cell loss by non-viable exclusion prior to 16-h time point signal measurement. This was tested by challenging cells with 10 µM of each filipin compound, staurosporine, or vehicle control and sampling cell populations across time points to measure cCAS3 induction and permeation by flow cytometry. At each timepoint, cells were stained with Ax700 to detect non-viable cells prior to fixing, then permeabilized with methanol and stained with fluorescent cCAS3 antibody to measure apoptosis. By 60 min, 80% or more of cells treated with **1,** **2**, and **4** exhibited cell population shifts towards the Ax700 positive, non-viable phenotype independent of cCAS3 activation (Fig. 5a). This phenomenon was observed at all timepoints measured after 60 min (Fig. 5 and S8). Compound **3**; however, did not induce significant cCAS3 or non-viable marker shifts at any timepoint which indicated hydroxylation of the 3’ carbon on a four-carbon pendant alkyl chain prevents filipin-induced cell permeation.

These observations were confirmed with a dimetric fluorescent microscopy assay that utilized propidium iodide (PI), a red fluorescent DNA intercalator that only penetrates cells with compromised cell membranes, and quinacrine, a green fluorescent intracellular ATP detection agent that is lost following membrane permeation [31, 32]. MV-4-11 cells treated with 10 µM of **3** presented robust quinacrine staining and few PI positive cells comparable to vehicle control (Fig. 5b, c) Alternatively, 10 μM of **1,** **2**, and **4** all permeabilized cell membranes as confirmed by PI positive staining and subsequent loss of quinacrine staining (Fig. 5b, c). Staurosporine positive control was positive for cCAS3 induction, PI infiltration, and quinacrine signal loss while vehicle control remained cCAS3 and PI negative and quinacrine signal positive.

(Fig. S9). In combination, flow cytometry and fluorescent microscopy confirmed that diminished cell populations observed in MAM are consistent with the bioactivity of **1** and **4**. The observed cytotoxicity of **2** (Fig. 5) was unexpected and most likely failed to deplete viable cells in MAM because of insufficient fractionated well concentrations (Fig. 3b and S3). Moreover, all compounds share identical polyene, macrolide chemical scaffolds with varied alkane pendant lengths. Only compound **3**, which carries a hydroxyl group on C 3’, was found to be non-cytotoxic (Figs. 4, 5).

## Discussion

This work describes a workflow for microbial natural product discovery based on single cell responses to extracts, fractions, and compounds using fluorescent cell barcoding to multiplex wells and multiple marker fluorophore-tagged antibodies to multiplex functional readouts per cytometric analysis. This study demonstrated the metabolomic potential of *K. psammotica* ssp*. carrieae* to synthesize a family of filipin molecules in a culture condition-dependent fashion. MAP rapidly identified cytotoxic extracts against the MV-4-11 acute myeloid leukemia cell line. The active extracts were then fractionated using MAM to identify secondary metabolites responsible for observed mammalian cell phenotypes and ultimately informed the decision to isolate filipin compounds by bioactivity and shared UV spectra.

The MAP and MAM workflow outlined in this work used a set of general cell death and injury markers to detect compound-induced changes that integrate a variety of possible biological mechanisms. Cellular responses were judged as the arcsinh ratio or arcsinh ratio of percent in-gate vs. vehicle control of cCAS3 and γH2AX marker signals [14] in MAP and MAM assays of *K. psammotica* ssp*. carrieae* extracts and metabolomic arrays, respectively. This motivated the investigation of the degree of cCAS3-dependency of the cytotoxicity of isolated filipins. Microscopy and flow cytometry demonstrated that filipins do not induce cCAS3-mediated apoptosis and data were consistent with the known pore-forming depolarization mechanism of filipin-type polyene macrolides. These data suggest that binding of **1,** **2**, and **4** nonspecifically to sterols can disrupt cell membranes sufficiently enough for penetration of the vital dye Ax700 and is consequently reflected in diminished viable cell populations in wells containing filipins at sufficient concentrations [33, 34]. These observations support the conclusion that increases in general cell injury markers such as cCAS3 and γH2AX via cytometry may be observed for many cytotoxic compounds. Moreover, cCAS3 dependency on cytotoxic compounds, for example the cCAS3 signaling apoptosis-inducing internal plate control compound staurosporine used in MAP and MAM, may be readily distinguished from membrane disrupting cytotoxic discovery leads such as the filipins using traditional apoptosis characterization methods described herein.

The MAP and MAM assays are enabled by the inherent efficiencies of fluorescent cell barcoding. This strategy enabled multiplexing of 48 unique conditions to simultaneously detect six biometric readouts (viability, cCasp3, γ-H2AX, p-S6, p-Hh3, and DNA content). Fluorescent cell barcoding also afforded a robust staining protocol that decreased fluorescent antibody reagent consumption 48-fold per antibody, ensured consistent staining across well samples, and reduced instrumentation time [14]. Together, these factors accrue an estimated 41-fold decrease in costs per flow cytometry run. Moreover, MAM integrated multidimensional chromatographic, mass spectrometric and UV data to yield well-defined metabolomic fractions for independent evaluation of bioeffector potential. These quickly informed decisions to isolate candidate bioactive natural products from complex extracts.

The workflow enabled by MAP and MAM with automated data analysis using the DebarcodeR algorithm demonstrated a generalizable platform for identification of optimal production conditions and discovery of microbial secondary metabolites active against cancer cells. Previous studies show that secondary metabolites inducing cell death and/or injury pathways can be readily detected via MAM workflows. In addition to control compounds described herein, we have observed marker-dependent activities in the activity metabolomics chromatograms in both cell lines and primary cells revealing glycomacrolide apoptolidin A, macrolactam ciromicins, and anthracyclines, to name a few [14]. Discovering interesting phenotypes as part of isolation can stimulate follow up mechanistic studies [35]. The assay phenotype engendered by filipins identified in this case study demonstrates that membrane-targeting metabolites such as filipins, other polyene antifungals and membrane-targeting compounds may not target intracellular processes prior to reaching a critical concentration disrupting cell membranes, and therefore may not perturb canonical markers of cell injury and regulated cell death prior to abrupt cell depolarization. As a significant number of secondary metabolites exert their effect via targeting membranes, many such compounds may engender non-specific phenotypes in cell death and injury pathways, and this work aids in delineating this type of mechanism prior to isolation.

## Supplementary information

Supplement 3

Supplement 1

Supplement 2
