# Supplementary material for: Multiplexed activity metabolomics for isolation of filipin macrolides from a hypogean actinomycete
Source: J Antibiot (Tokyo). 2024 Dec 6;78(2):78–89. doi: 10.1038/s41429-024-00792-6 (PMC11769839; doi:10.1038/s41429-024-00792-6)

Supplement 3 text description of file

Response time course to filipin challenge in MV-4-11 cell line…………………………………S8

Flow cytometry and fluorescent microscopy controls…………………………………………....S9


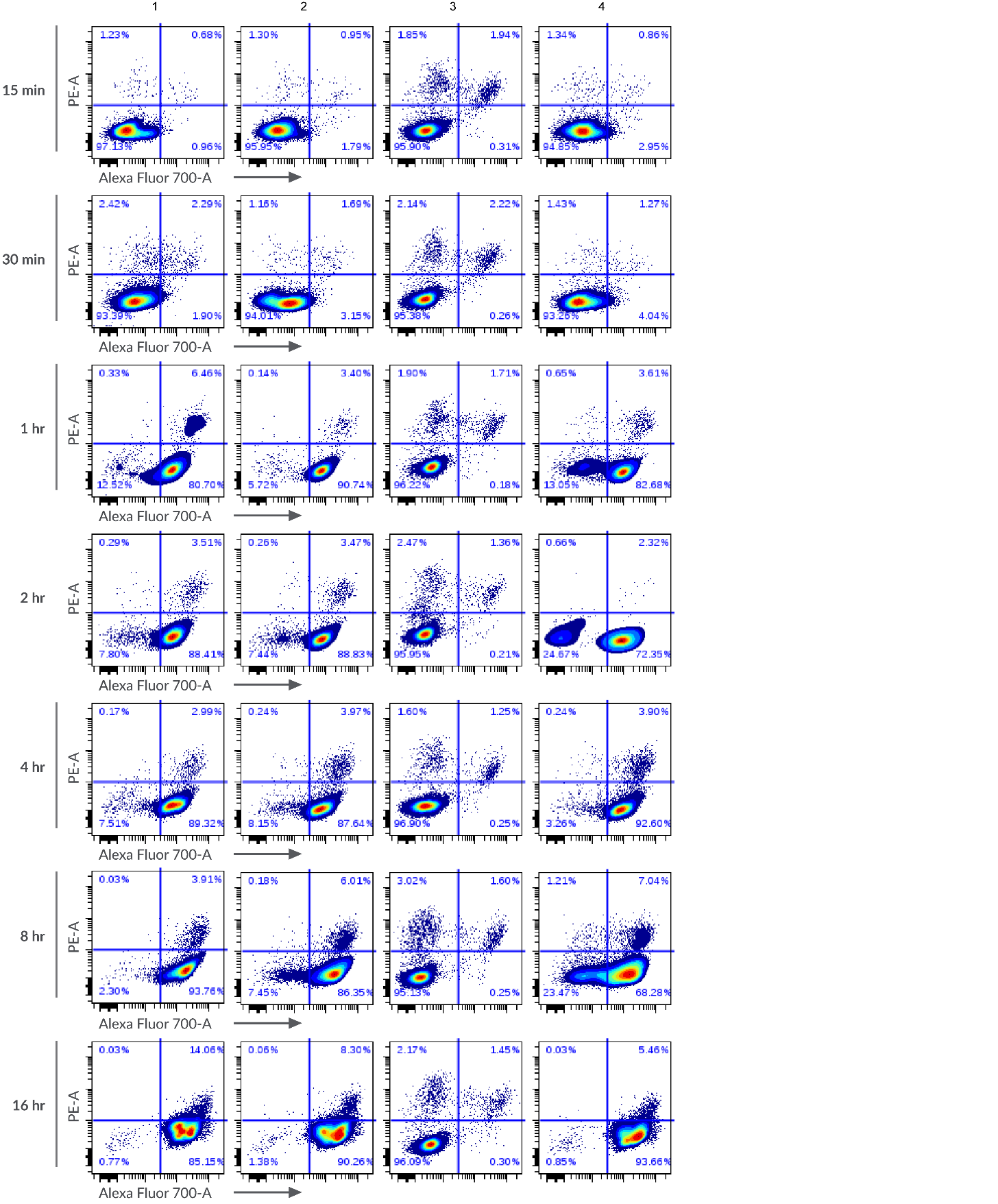


**Fig. S8**: Response time course to filipin challenge in MV-4-11 cell line. Filipin II (**1**), chainin (**2**), and filipin IX (**4**) induce cCAS3 independent death as early as 1 hour. Filipin XV (**3**) is non-toxic.


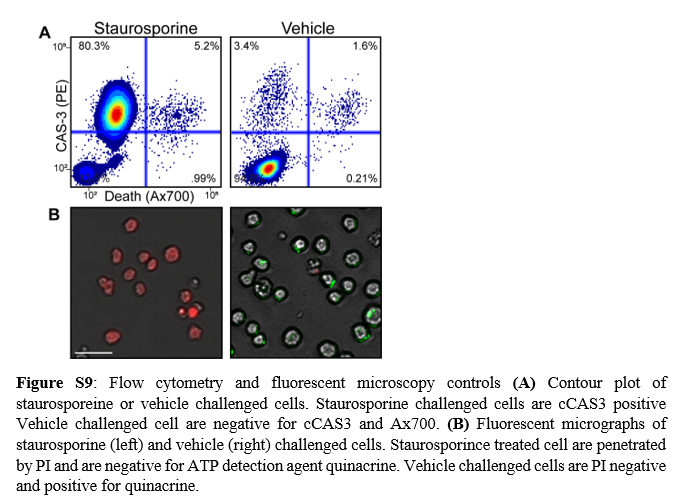

Supplement: Supplementary file 1 — Supplement 3 [file 41429_2024_792_MOESM1_ESM.docx]
