# Supplementary material for: Multiplexed activity metabolomics for isolation of filipin macrolides from a hypogean actinomycete
Source: J Antibiot (Tokyo). 2024 Dec 6;78(2):78–89. doi: 10.1038/s41429-024-00792-6 (PMC11769839; doi:10.1038/s41429-024-00792-6)

Supplement 1 text description of file

*K. psammotica ssp carrieae* images……………………………………………………………...S1

MAM alignment for filipin family members…………………………………………………….S2

MAM alignment of extracted ion currents of filipin compounds with plate well……………….S3


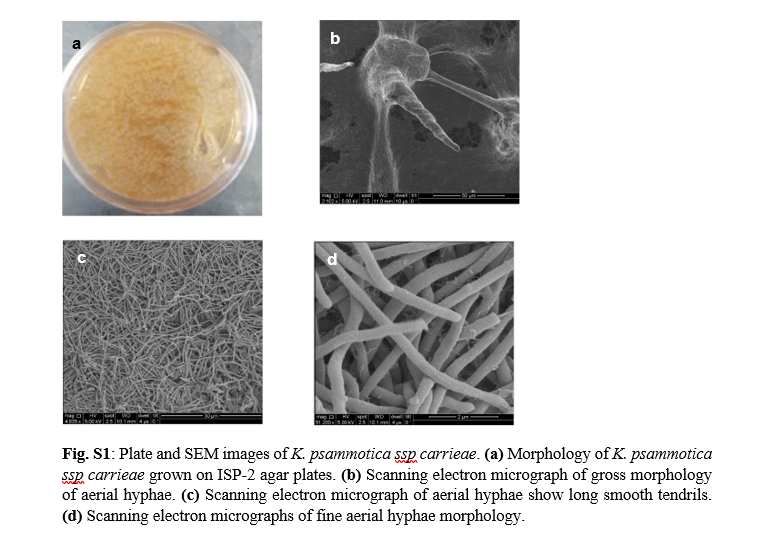


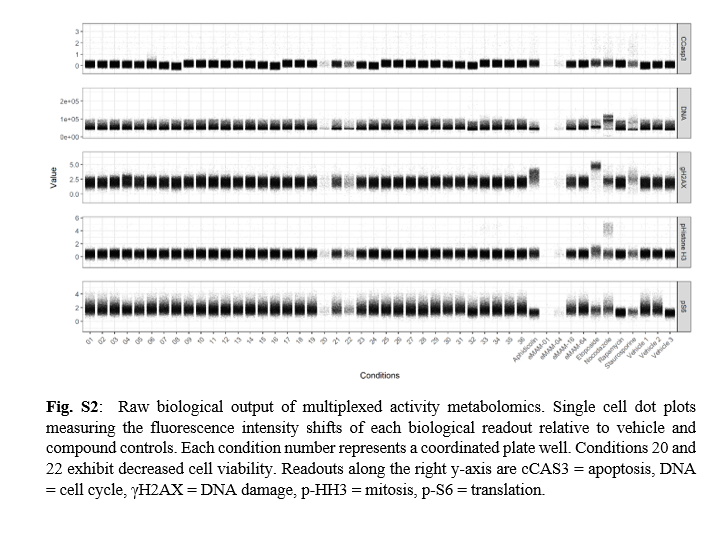


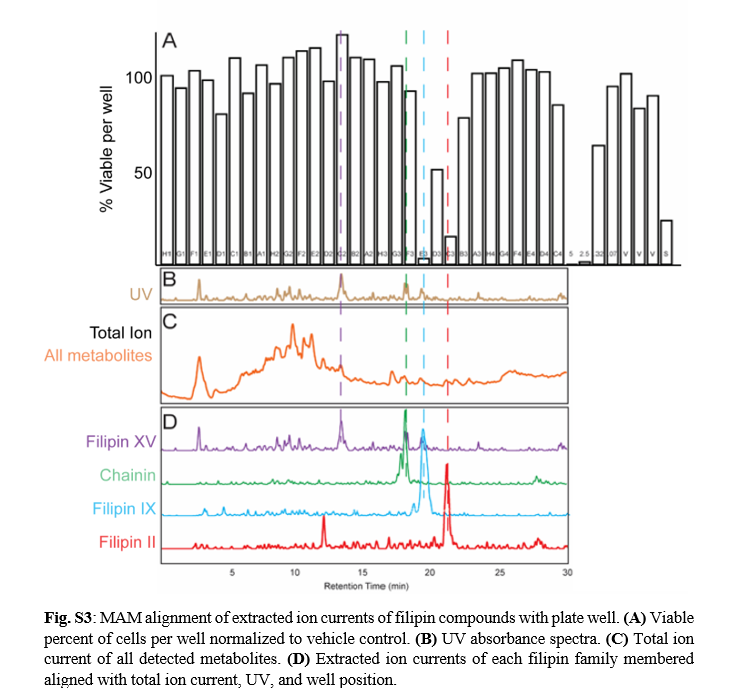

Supplement: Supplementary file 2 — Supplement 1 [file 41429_2024_792_MOESM2_ESM.docx]
