# Supplementary material for: Multiplexed activity metabolomics for isolation of filipin macrolides from a hypogean actinomycete
Source: J Antibiot (Tokyo). 2024 Dec 6;78(2):78–89. doi: 10.1038/s41429-024-00792-6 (PMC11769839; doi:10.1038/s41429-024-00792-6)
Supplement: Supplementary file 3 — Supplement 2 [file 41429_2024_792_MOESM3_ESM.docx]

Supplement 2 text description of file

Filipin II ^1^H NMR………………………………………………………………………………..S4

Filipin II correlation table………………………………………………………………………..T1

Chainin ^1^H NMR…………………………………………………………………………………S5

Chainin correlation table…………………………………………………………………………T2

Filipin XV ^1^NMR……………..………………………………………………………….………S6

Filipin XV correlation able………………………………………………………………………T3

Filipin IX ^1^H MR………………………..…………………………………………………….….S7

Filipin IX correlation table………………….……………….……………………………….…..T4

**Fig. S4**: ^1^H NMR spectra of filipin II. Analysis performed in methanol-d4.

**Table S1**: NMR correlation table for filipin II. Analysis performed in methanol-d4.

| Pos. | Proton | Carbon | COSY | HMBC |
| --- | --- | --- | --- | --- |
| 1 | N/A | 174 |  | 2, 27 |
| 2 | 2.35 (m) | 52.9 | 3, 1' |  |
| 3 | 3.85 (m) | 72 | 2,4 | 1 |
| 4 | 1.43 (m) | 41.1 | 3, 5 |  |
| 5 | 4.04 (m) | 71.8 |  |  |
| 6 | 1.34-1.52 (m, 2H) | 41-43.5 |  |  |
| 7 | 4.04 (1H, m) | 71.8 |  |  |
| 8 | 1.34-1.52 (m, 2H) | 41-43.5 |  |  |
| 9 | 4.04 (1H, m) | 71.8 |  |  |
| 10 | 1.34-1.52 (m, 2H) | 41-43.5 |  |  |
| 11 | 4.04 (1H, m) | 69.5 |  |  |
| 12 | 1.75, 1.49 (2H, m) | 43.6 |  |  |
| 13 | 3.35 (1H, m) | 66.1 | 14, 12 |  |
| 14 | 1.76, 1.93 (2H, m) | 41.2 | 15, 13 |  |
| 15 | 4.19 (1H, dd, 4.3, 10.3) | 74.2 | 14 |  |
| 16 | N/A | 139.4 |  | 29 |
| 17 | 6.10 (1H, d, 11.2) | 126.6 | 18, 29 | 29, 15, 19 |
| 18 | 6.54 (1H, dd, 11.3, 14.4) | 128.1 | 19, 17 |  |
| 19 | 6.36 (1H, m) | 132.7 | 18 |  |
| 20 | 6.35-6.43 (1H, m) | 132.7 |  |  |
| 21 | 6.35-6.43 (1H, m) | 132.7 |  |  |
| 22 | 6.35-6.43 (1H, m) | 132.7 |  |  |
| 23 | 6.34 (1H, m) | 132.7 |  |  |
| 24 | 6.41 (1H, m) | 132.3 | 25, 23 | 25, 23 |
| 25 | 5.95 (1H, dd, 5.8, 14.8) | 132.7 | 26, 24 | 26, 24 |
| 26 | 4.04 (m) | 72.8 |  |  |
| 27 | 4.86 (1H, m) | 73.1 | 28, 26 | 1, 26 |
| 28 | 1.33 (m, 3H) | 16.9 | 27 |  |
| 29 | 1.80 (3H, s) | 10.1 | 17 | 15, 17, 16 |
| 1' | 1.61, 1.72 (m) | 28.8 | 2 |  |
| 2' | 1.25-1.34 (m, 2H) | 26.9 |  |  |
| 3' | 1.35 (2H, m) | 28.8 |  |  |
| 4' | 1.31 (2H, m) | 31.4 |  |  |
| 5' | 1.39 (2H, m) | 22.2 |  |  |
| 6' | 0.8 (3H, t, 7.1) | 13 | 5' | 5', 4' |

**Fig. S5**: ^1^H NMR spectra of chainin. Analysis performed in methanol-d4.

**Table S2**: NMR correlation table for chainin. Analysis performed in methanol-d4.

| Pos | Proton | Carbon | COSY | HMBC |
| --- | --- | --- | --- | --- |
| 1 | N/A | 174 |  | 2, 3, 27 |
| 2 | 2.35 (m) | 52.9 | 3, 1' | 1, 1' |
| 3 | 3.85 (m) | 72 | 2, 4 | 1, 5, 1' |
| 4 | 1.43 (m) | 41.1 | 5 |  |
| 5 | 4.04 (m) | 71.8 |  |  |
| 6 | 1.34-1.52 (m, 2H) | 41-43.5 |  |  |
| 7 | 4.04 (1H, m) | 71.8 |  |  |
| 8 | 1.34-1.52 (m, 2H) | 41-43.5 |  |  |
| 9 | 4.04 (1H, m) | 71.8 |  |  |
| 10 | 1.34-1.52 (m, 2H) | 41-43.5 |  |  |
| 11 | 4.04 (1H, m) | 69.5 |  |  |
| 12 | 1.75, 1.49 (2H, m) | 43.6 |  |  |
| 13 | 3.35 (1H, m) | 66.1 | 12, 14 |  |
| 14 | 1.76, 1.93 (2H, m) | 41.2 | 13, 15 |  |
| 15 | 4.19 (1H, dd, 4.3, 10.1) | 74.2 | 14 |  |
| 16 | N/A | 139.4 |  | 18, 29 |
| 17 | 6.10 (1H, d, 11.3) | 126.6 | 18, 29 | 15, 19, 29 |
| 18 | 6.54 (1H, dd, 11.2, 14.2) | 128 | 19, 17 | 16, 17, 19 |
| 19 | 6.36 (1H, m) | 132.7 | 18 |  |
| 20 | 6.35-6.43 (1H, m) | 132.7 |  |  |
| 21 | 6.35-6.43 (1H, m) | 132.7 |  |  |
| 22 | 6.35-6.43 (1H, m) | 132.7 |  |  |
| 23 | 6.34 (1H, m) | 132.7 |  |  |
| 24 | 6.41 (1H, m) | 132.3 | 25, 23 | 25, 23 |
| 25 | 5.95 (1H, dd, 5.9, 15.1) | 132.7 | 26, 24 | 26, 24 |
| 26 | 4.04 (m) | 72.8 |  |  |
| 27 | 4.86 (1H, m) | 73.1 | 26, 28 | 1, 25, 26 |
| 28 | 1.33 (m, 3H) | 16.9 | 27 | 26 |
| 29 | 1.80 (3H, s) | 10.1 | 17 | 13, 15, 16, 17 |
| 1' | 1.60, 1.72 (m) | 28.8 |  |  |
| 2' | 1.35 (2H, m) | 31.2 |  |  |
| 3' | 1.39 (2H, m) | 22.2 |  |  |
| 4' | 0.8 (3H,m) | 13 | 3' | 3', 2', 1' |

**Fig. S6**: ^1^H NMR spectra of filipin XV. Analysis performed in methanol-d4.

**Table S3**: NMR correlation table for filipin XV. Analysis performed in methanol-d4.

| Pos. | Proton | Carbon | COSY | HMBC |
| --- | --- | --- | --- | --- |
| 1 | N/A | 173.7 |  | 27 |
| 2 | 2.35 (m) | 52.9 | 3, 1' | 1, |
| 3 | 3.85 (m) | 72 | 2, 4 |  |
| 4 | 1.43 (m) | 41.1 | 3, 5 |  |
| 5 | 4.04 (m) | 71.8 |  |  |
| 6 | 1.34-1.52 (m, 2H) | 41-43.5 |  |  |
| 7 | 4.04 (1H, m) | 71.8 |  |  |
| 8 | 1.34-1.52 (m, 2H) | 41-43.5 |  |  |
| 9 | 4.04 (1H, m) | 71.8 |  |  |
| 10 | 1.34-1.52 (m, 2H) | 41-43.5 |  |  |
| 11 | 4.04 (1H, m) | 69.5 |  |  |
| 12 | 1.73, 1.49 (2H, m) | 43.6 |  |  |
| 13 | 3.35 (1H, m) | 66.1 | 12, 14 |  |
| 14 | 1.77, 1.93 (2H, m) | 41.2 | 13, 15 |  |
| 15 | 4.19 (1H, dd, 4.3, 10.1) | 74.2 | 14 |  |
| 16 | N/A | 139.4 |  | 16 |
| 17 | 6.10 (1H, d, 11.3) | 126.6 | 18 | 15, 19, 29 |
| 18 | 6.54 (1H, dd, 11.2, 14.2) | 128 | 17, 19 | 19 |
| 19 | 6.36 (1H, m) | 132.7 |  |  |
| 20 | 6.35-6.43 (1H, m) | 132.7 |  |  |
| 21 | 6.35-6.43 (1H, m) | 132.7 |  |  |
| 22 | 6.35-6.43 (1H, m) | 132.7 |  |  |
| 23 | 6.34 (1H, m) | 132.7 |  |  |
| 24 | 6.41 (1H, m) | 132.3 | 23, 25 | 23, 25 |
| 25 | 5.95 (1H, dd, 5.9, 15.1) | 132.7 | 24, 26 | 24, 26 |
| 26 | 4.04 (m) | 72.8 |  |  |
| 27 | 4.86 (1H, m) | 73.1 | 26, 28 | 1, 25, 26 |
| 28 | 1.33 (m, 3H) | 16.9 | 27 | 27, 26 |
| 29 | 1.80 (3H, s) | 10.1 | 17 | 15, 16, 17 |
| 1' | 1.62 (m) | 30.3 |  |  |
| 2' | 1.42 (2H, m) | 36.2 |  |  |
| 3' | 3.71 (1H, m) | 66.8 | 4', 2' |  |
| 4' | 1.16 (3H,m) | 22.1 | 3' | 3', 2' |

**Fig. S7**: ^1^H NMR spectra of filipin IX. Analysis performed in methanol-d4.

**Table S4**: NMR correlation table for filipin IX. Analysis performed in methanol-d4.

| Pos. | Proton | Carbon | COSY | HMBC |
| --- | --- | --- | --- | --- |
| 1 | N/A | 174 |  | 27 |
| 2 | 2.35 (m) | 52.9 | 3, 1' |  |
| 3 | 3.85 (m) | 72 | 2, 4 | 1, 5 |
| 4 | 1.43 (m) | 41.1 | 5 |  |
| 5 | 4.04 (m) | 71.8 |  |  |
| 6 | 1.34-1.52 (m, 2H) | 41-43.5 |  |  |
| 7 | 4.04 (1H, m) | 71.8 |  |  |
| 8 | 1.34-1.52 (m, 2H) | 41-43.5 |  |  |
| 9 | 4.04 (1H, m) | 71.8 |  |  |
| 10 | 1.34-1.52 (m, 2H) | 41-43.5 |  |  |
| 11 | 4.04 (1H, m) | 69.5 |  |  |
| 12 | 1.75, 1.49 (2H, m) | 43.6 |  |  |
| 13 | 3.35 (1H, m) | 66.1 | 14, 12 |  |
| 14 | 1.76, 1.93 (2H, m) | 41.2 | 13, 15 |  |
| 15 | 4.19 (1H, dd, 4.3, 10.1) | 74.2 | 14 |  |
| 16 | N/A | 139.4 |  | 29 |
| 17 | 6.10 (1H, d, 11.3) | 126.6 | 29, 18 | 29, 15, 19 |
| 18 | 6.54 (1H, dd, 11.2, 14.2) | 128 | 19, 17 | 19 |
| 19 | 6.36 (1H, m) | 132.7 | 18 |  |
| 20 | 6.35-6.43 (1H, m) | 132.7 |  |  |
| 21 | 6.35-6.43 (1H, m) | 132.7 |  |  |
| 22 | 6.35-6.43 (1H, m) | 132.7 |  |  |
| 23 | 6.34 (1H, m) | 132.7 |  |  |
| 24 | 6.41 (1H, m) | 132.3 | 25, 23 | 25, 23 |
| 25 | 5.95 (1H, dd, 5.8, 15.1) | 132.7 | 26, 24 | 26, 24 |
| 26 | 4.04 (m) | 72.8 |  |  |
| 27 | 4.86 (1H, m) | 73.1 | 28, 26 | 1, 26, 25 |
| 28 | 1.33 (m, 3H) | 16.9 | 27 |  |
| 29 | 1.80 (3H, s) | 10.1 | 17 | 15, 17, 16 |
| 1' | 1.61, 1.72 (m) | 28.8 |  |  |
| 2' | 1.35 (2H, m) | 29 |  |  |
| 3' | 1.31 (2H, m) | 31.4 |  |  |
| 4' | 1.39 (2H, m) | 22.2 |  |  |
| 5' | 0.8 (3H,m) | 13 | 4' | 4', 3' |
